# From Food to Offspring Down: Tissue-Specific Discrimination and Turn-Over of Stable Isotopes in Herbivorous Waterbirds and Other Avian Foraging Guilds

**DOI:** 10.1371/journal.pone.0030242

**Published:** 2012-02-01

**Authors:** Steffen Hahn, Bethany J. Hoye, Harry Korthals, Marcel Klaassen

**Affiliations:** 1 Swiss Ornithological Institute, Sempach, Switzerland; 2 Department Animal Ecology, The Netherlands Institute of Ecology (NIOO-KNAW), Wageningen, The Netherlands; 3 Department of Aquatic Ecology, The Netherlands Institute of Ecology (NIOO-KNAW), Wageningen, The Netherlands; 4 Centre for Integrated Ecology, Deakin University, Geelong, Victoria, Australia; Texas A&M University-Corpus Christi, United States of America

## Abstract

Isotopic discrimination and turn-over are fundamental to the application of stable isotope ecology in animals. However, detailed information for specific tissues and species are widely lacking, notably for herbivorous species. We provide details on tissue-specific carbon and nitrogen discrimination and turn-over times from food to blood, feathers, claws, egg tissues and offspring down feathers in four species of herbivorous waterbirds. Source-to-tissue discrimination factors for carbon (δ^13^C) and nitrogen stable isotope ratios (δ^15^N) showed little variation across species but varied between tissues. Apparent discrimination factors ranged between −0.5 to 2.5‰ for δ^13^C and 2.8 to 5.2‰ for δ^15^N, and were more similar between blood components than between keratinous tissues or egg tissue. Comparing these results with published data from other species we found no effect of foraging guild on discrimination factors for carbon but a significant foraging-guild effect for nitrogen discrimination factors.

Turn-over of δ^13^C in tissues was most rapid in blood plasma, with a half-life of 4.3 d, whereas δ^13^C in blood cells had a half-life of approximately 32 d. Turn-over times for albumen and yolk in laying females were similar to those of blood plasma, at 3.2 and 6.0 d respectively. Within yolk, we found decreasing half-life times of δ^13^C from inner yolk (13.3 d) to outer yolk (3.1 d), related to the temporal pattern of tissue formation.

We found similarities in tissue-specific turn-over times across all avian species studied to date. Yet, while generalities regarding discrimination factors and tissue turn-over times can be made, a large amount of variation remains unexplained.

## Introduction

The stable isotope composition of a consumer's tissue is generally assumed to reflect the isotopic composition of its assimilated diet at the time of tissue synthesis [1]. As such, the specific composition of stable isotopes in consumer tissues are commonly used to address questions related to foraging ecology, e.g. the determination of foraging niches [2], or an individuals' position within food webs [3]. Moreover, geographically specific isotope patterns archived in animals tissues allows for spatial-temporal assignments of individuals and for tracking of migration [4]. However, such studies fundamentally rely on *a priori* knowledge of (1) how stable isotopes discriminate between the original source, either food or body reserve, and the target tissue; and (2) the speed at which a change in dietary isotopic regime is mirrored in the focal tissue, known as tissue turn-over time. That is, how reliably and rapidly animals incorporate the isotopic composition of the resources they use [5].

Discrimination (ΔX) essentially describes the specific change of the isotopic composition of the chemical element X, either enrichment or depletion, occurring between source and tissue, such that ΔX = δX_tissue_−δX_source_ [6]. Herein, each δ notation gives a difference measurement made relative to a standard δX = [(R_sample_/R_standard_−1)]×1000 [7]. By considering the general composition of soure and tissue and ignoring component specific assimilation efficiencies or tissue specific nutrient allocation [8], an ‘apparent discrimination factor’ can be derived from the difference between isotope data of tissue and source [6]. Such discrimination factors have been shown to vary according to target tissue [9], diet protein content [10] [11], and the nutritional state of the organism from which the tissue is sampled [12].

Tissue turn-over time is the period required to incorporate a specific isotope pattern from source into body tissue. Turnover time, therefore, determines the temporal window during which changes in the isotopic composition of an animal's diet can be discerned [5]. Turn-over times, like discrimination factors, vary between different tissues and depend on the speed of tissue renewal [13]. Metabolically active tissues, such as blood components, liver, and muscle, represent a moving window of information on diet composition [4]. For example, blood cells and blood plasma show rapid turn-over and therefore an archive on the scale of days to a few weeks only [14], whereas bone collagen shows much longer turnover times. In inert tissues, such as hair, claws and feathers, isotopic information is retained, but only reflects the resources incorporated during the discrete time interval in which that tissue was grown [4]. Such tissues, therefore, afford insights into the forgoing months to years [14] [15]. Finally, the speed of tissue formation co-affects its final isotope composition. For example, when moulting birds travel between different isotopic regions the isotope composition may differ both between and within feathers [16].

Conclusions derived from isotope measures fundamentally rely on appropriate discrimination factors, and detailed knowledge of turn-over [17] [18]. Small errors in the discrimination and turnover values used can have substantial ramifications for the interpretation of isotopic measurements, and yet many isotopic studies in birds rely on a limited number of investigations into discrimination and turn-over times in avian tissues, especially for tissues other than blood [19] [15] [6] [20]. Indeed, many studies have used discrimination factors derived from inter-specific means or single values measured in other species or tissues [11] [9]. Discrimination factors have been shown to vary according to target tissue and taxonomic grouping [9], dietary protein quantity and quality [10] [6] [12] – with contradictory results depending on the nutritional state of the birds [21], temperature [22], and mode of nitrogen excretion [23]. However, the strength and direction of these relationships have proven equivocal, largely because the mechanistic basis for such differences are not understood [18]. As such, extrapolation of any such relationship to estimate discrimination for a study lacking empirical values remains problematic [24], and has prompted a renewed call for additional experimental data on discrimination and turnover [5].

Once discrimination factors and turn-over times are known, however, stable isotope compositions of tissues carry valuable information about food or habitat use over various time frames. Here, we provide critical baseline information about tissue-specific discrimination and turn-over of stable carbon (δ^13^C) and stable nitrogen isotope ratios (δ^15^N) in herbivorous duck, geese and swan species (Anatidae); a group of birds that are often used as model organisms but for which the prerequisite isotopic data are still lacking. We assess isotopic patterns from food, different blood tissues, body feathers, egg tissue, and finally chick down feathers, to provide unique data covering the annual cycle in these species. Moreover, we compare these data with data compiled from the literature to assess generalizations across avian foraging guilds and a range of body masses.

## Methods

### Ethics statement

The experiments in the Netherlands were run under animal ethics permissions CL03.01 and CL09.04 from the Royal Dutch Academy of Arts and Sciences (KNAW). Permission for field work at Svalbard was given by the governor of Svalbard (2006/00482-4 a.522.01).

### Source-tissue discrimination

In the first part of our study we examined discrimination of δ^15^N and δ^13^C from food to various body tissues, including blood plasma (plasma), red blood cells (cells), feathers (primaries and back feathers) and claws in Bewick's Swans (*Cygnus columbianus bewickii*, n = 9) and European Mallards (*Anas platyrhynchos*, n = 9). Birds were held in outdoor aviaries for >2 years and fed a diet resembling that consumed in the wild, containing 50% grain mixture and 50% chicken mash (HAVENS Voeders, Maashees, the Netherlands). Diet composition was constant during the experiment, but origin of the grains supplied may have varied. The isotopic composition of the diet was −21.46±0.39‰ for δ^13^C (SD) and 1.95±0.45‰ for δ^15^N (SD); C/N ratio by atomic mass was 20.3/1 (two random samples of the main components of the food mix). Blood samples were collected from the tarsal vein of nine individuals of each species (5 males and 4 females) and centrifuged (7 g; 10 minutes) immediately to separate plasma from blood cells. We also collected a sample from the tip of the first primary feather, a complete feather from the back, and a sample (∼1 mm in length) from the tip of the inner-most claw. All blood cells (in 70% ethanol) and plasma samples were stored at −20°C until analysis.

Second, we quantified discrimination of δ^15^N and δ^13^C from food to egg tissue and then to chick down feathers in wild populations of Pink-footed Geese (*Anser brachyrhynchos*) and Barnacle Geese (*Branta leucopsis*). Study populations winter in north-western Europe and migrate to their breeding grounds in Svalbard with staging sites at the Norwegian coast. Herein the food at staging and breeding sites are isotopically distinct [25]. To quantify discrimination from food to eggs we used droppings from Pink-footed Geese as proxies for food, because droppings reflect the quantitative composition of ingested food in geese [26]. Grass and moss as main diet components contain a large portion of non-digestible fibre, which will not be chemically modified during gut passage and are excreted afterwards. Thus, data from droppings mainly represents the isotope composition of fibre. However, protein content of bulk grass and dropping can differ due to (partly) digestion of the proteins [27], and thus, can affect δ^15^N (mainly protein) but rarely affects δ^13^C (mainly fibre). We collected droppings (with uric acid caps removed) at the two prime staging areas in Norway in 2004 and 2006. Isotope composition in droppings (n = 44) was −29.3±0.76‰ and 5.4±1.56‰ for δ^13^C and δ^15^N, respectively; C/N atomic mass ratio 15.5/1 [25]. Values for yolk isotopic composition were obtained from well-developed follicles of four female Pink-footed Geese shot at Hornsund (Svalbard) in 2004 immediately after crossing the Barents Sea; precisely on arrival from spring migration. To quantify discrimination from egg components to offspring down we collected 10 eggs of Pink-footed Geese and 9 eggs of Barnacle Geese on Svalbard in 2006 and 2007 ([25], Klaassen et al. unpublished data). From each egg, we collected samples of albumen, yolk and embryo feathers to get egg specific discrimination factors. The estimated mean age of embryos was 19±2.7 d. All samples were stored in 70% ethanol at −20°C until analysis.

### Turn-over in blood and egg tissues

We experimentally measured turn-over time of blood components and egg tissues in European Mallards by switching between two diets that differed in δ^13^C composition. Such switches can occur in waterfowl feeding on isotopically distinct resources throughout the annual cycle. In ducks, final egg formation lasts ∼8 days, with yolk deposition occurring during a 6 d period of rapid follicular growth followed by the segregation of albumen 1–2 days before the egg is laid [28]. If the isotopic composition of diet changes during egg formation, we would expect longer or similar turn-over times in yolk compared to blood plasma, because the latter acts as main carrier of yolk precursors. Because yolk is deposited in concentric layers during rapid follicular growth [29], we anticipate a temporal pattern of isotope composition within the egg: the oldest, inner-most yolk would have longer turn-over times and therefore the isotopic composition of the previous diet, while the youngest, outer-most yolk would have the shortest turn-over time and an isotopic composition more similar to that of the new diet.

Six mallards were held in outdoor aviaries in pairs, and fed a diet based on C4 plants (maize) or C3 plants (wheat). Both diets were composed of 67% wheat or maize, 22% soybean and 2% of sunflower seed, flax seed, millet, buckwheat and peanut. Ingredients were coarsely ground but not compressed into pellets. The isotopic composition of the C4-based diet was −16.2±1.52‰ for δ^13^C and 4.4±0.84‰ for δ^15^N, with a C/N ratio by atomic mass of 16.4/1. The isotopic composition of the C3-based diet was −26.9±0.41‰ for δ^13^C and 2.1±0.43‰ for δ^15^N, with a C/N ratio of 14.4/1. After 40–47 d of acclimatization on the C4 diet, food was switched to the C3 diet. We collected blood samples before and after the diet switch, and collected eggs laid by females during the experiments. Blood samples were centrifuged immediately for plasma/blood cell separation. Eggs were boiled and a sample of albumen and samples from the inner (earliest) segregated yolk, intermediate and outer (latest) segregated yolk were collected. All samples were stored at −20°C until analysis.

### Stable isotope analysis

Food samples from the diet-tissue discrimination experiment were air-dried at 60°C for two days and ground to fine powder using an analytical mill (mesh size <1 mm). The keratinous samples (feathers and claws) were cleaned with hexane to remove any contamination, and air-dried under a fume hood. All other samples were freeze-dried for two days. We removed lipids from a sub-sample of whole yolk by extraction with chloroform-methanol (2/1 by volume). For each tissue (and food sample), sub-samples of 200–500 µg were analyzed for δ^15^N (‰ difference from the ^15^N/^14^N ratio in atmospheric N_2_) and for δ^13^C (‰ difference from ^13^C/^12^C ratio in Vienna PeeDee limestone) in a HEKAtech EuroEA elemental analyzer coupled on-line through a Finnigan con-flo interface to a Finnigan Delta S isotope ratio mass spectrometer. Reproducibility based on replicate measurements (n = 144) of a casein standard during the period of measurements was 0.14‰ (SD) for δ^15^N and 0.13‰ (SD) for δ^13^C.

### Calculation of discrimination factors and turn-over time

We calculated apparent discrimination factors (ΔX) for carbon and nitrogen stable isotopes between source and tissue as ΔX*_st_* = δX*_tissue_*−δX*_source_*, wherein sources were bulk diets or egg tissues (for chick down feathers). To describe tissue specific turn-over after a diet switch we calculated exponential decay regression curves with *δ(t) = δ(∞)+a•e^−λ• t^* wherein *δ(t)* is the isotope signature of the specific tissue at time *t* (days) since diet switch, *δ(∞)* is the isotopic composition of the tissues in equilibrium with the new C3-diet, *a* is the difference between *δ(0)* and *δ(∞)*, and *λ* is the turn-over rate [17]. In addition, we calculated half-life times, the time required to halve the difference in isotopic composition between *δ(t)* and *δ(∞)*, as *t_50_ = ln(2)•λ^−1^*.

### Statistical analyses

Exponential decay regression curves were fitted using SigmaPlot11 (least square fit). All other statistical analyses were conducted in SPSS18.0. Data are presented as means ±SD, unless otherwise stated.

## Results

### Source-tissue discrimination

δ^13^C composition differed between body tissues (*F_4,87_* = 21.29, *P* = 0.001), but not between species (*F_1,87_* = 0.57, *P* = 0.45, Supporting Material Table S1). However, the interaction term showed significant differences between species and tissues (*F_4,87_* = 6.02, *P* = 0.001). There was a similar pattern for δ^15^N (species: *F_4,87_* = 0.47, *P* = 0.49; tissues: *F_4,87_* = 39.57, *P* = 0.001; interaction: *F_4,87_* = 4.44, *P* = 0.003, Supporting Material Table S1).

Accordingly, discrimination factors varied mainly between tissues (Table 1; Table 2) and, in a few cases, between species (Table 1). For carbon, blood cells showed negative Δ^13^C, whereas plasma and keratinous tissues showed positive ΔC (Table 1). For nitrogen, Δ^15^N were more similar among tissues, increasing from blood cells (Δ^15^N: 3.6) to primary feathers (Δ^15^N: 5.2, Table 1).

**Table 1:** Discrimination factors Δ δ^13^C and Δ δ^15^N between food and various tissues of four herbivorous Anatidae species.

Source	Tissue	Δ δ^13^C	t-test	Δ δ^15^N	t-test	n^x^, n^y^
Food to:	Plasma	0.3±0.52^A,B^	1.52	4.4±0.62^A,B^	4.69*	9, 9
	Blood cells	−0.5±0.62^A,B^	1.59	3.6±0.52^A,B^	0.71	9, 9
	Claw	0.4±0.58^A,B^	0.56	4.5±0.67^A,B^	1.09	9, 9
	Back feather	1.0±1.03^A,B^	2.91*	4.7±0.71^A,B^	1.06	9, 9
	Primary feather	0.9±0.73^A,B^	1.73	5.2±0.56^A,B^	1.87	9, 9
Dropping to:	Yolk	2.5±0.81^D^	-	2.8±2.73^D^	-	4
Yolk to:	Down feather	−1.1±0.44^C,D^	2.55*	1.9±0.51^C,D^	2.27*	9, 10
Albumen to:	Down feather	−0.9±0.61^C,D^	0.27	2.7±1.29^C,D^	1.17	9, 10

Source -tissue discrimination was measured experimentally in captive Bewick Swans *Cygnus columbianus bewickii* (A) and mallards *Anas platyrhynchos* (B); diet and egg tissue- feather discrimination was determined in free-living Barnacle Geese *Branta leucopsis* (C) and Pink-footed Geese *Anser brachyrhynchos* (D). Herein, droppings were used as proxies for ingested food. Between-species differences were examined with t-test,

*marks a difference at p-level<0.05, n^x^ gives sample size for the respective species. Individual species values are provided in Table S2.

**Table 2:** Statistical comparison of δ^13^C and δ^15^N between various tissues of individual herbivorous waterbirds.

Tissue	Isotope	Blood cells		Claw		Back feather		Primary feather	
		*t*	*p*	*t*	*p*	*t*	*p*	*t*	*p*
Plasma	C	8.74	0.001	−1.79	0.10	−3.52	0.003	−3.84	0.001
	N	7.16	0.001	0.14	0.89	−4.39	0.001	−6.45	0.001
Blood cells	C			−7.16	0.001	−6.81	0.001	−14.04	0.001
	N			−10.11	0.001	−10.20	0.001	−20.57	0.001
Claw	C					−1.66	0.12	−2.04	0.06
	N					−2.37	0.04	−9.53	0.001
Back feather	C							0.84	0.41
	N							−3.73	0.002

Tissues are blood plasma, blood cells, and keratinous tissues: claw, back feather and first primary feather from Bewick Swans (*Cygnus columbianus bewickii*, n = 9) and mallards (*Anas platyrhynchos*, n = 9). Data were pooled for paired t-tests.

In egg tisues, Δ*_food to yolk_* was 2.5 (δ^13^C) and 2.8 (δ^15^N), respectively for lipid-free yolk (Table 1). Lipid-free Δ*_yolk to chick down_* were negative, and differed between Barnacle Geese and Pink-footed Geese in both isotopes (for δ^13^C: *t_25_* = −2.27, p = 0.03; for δ^15^N: *t_25_* = −2.55, *P* = 0.02). However, Δ*_albumen to chick down_* was similar for both species (for δ^13^C: *t_17_* = −0.27, *P* = 0.79; for δ^15^N: *t_17_* = 1.17, *P* = 0.26).

### *Turn-over time*

**Figure 1:**
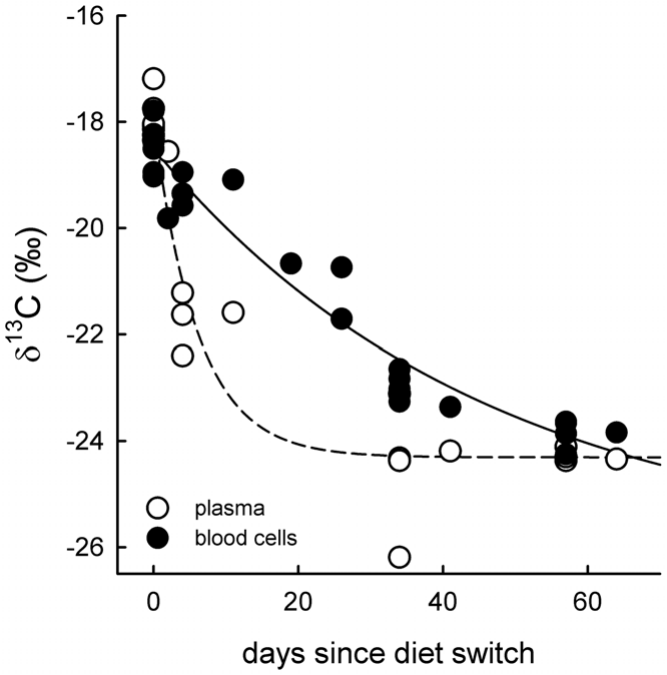
Turn-over of blood tissues. Turn-over of δ^13^C in plasma (unfilled circles, dashed line) and blood cells (filled circles, solid line) from mallards switched from C4-based to C3-based diets. For parameters of the exponential decay curves see Table 3.

**Figure 2:**
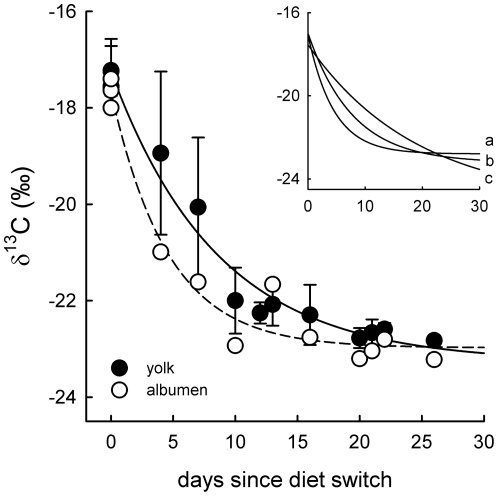
Turn-over of egg tissues. Turn-over of δ^13^C of albumen (unfilled circles, dashed line) and yolk (means ± SD, filled circles, solid line) in eggs laid by female mallards while switched from C4- to C3-based diet. Inset: Turn-over in different parts of yolk with a – inner, b – intermediate and c – outer yolk. For parameters of the exponential decay curves see Table 3.

In blood tissues, turn-over rate (*λ*) and half-life time (*t_50_*) of δ^13^C was 0.162±0.033 (SE) and 4.3 d respectively in plasma, and 0.022±0.007 (SE) and 31.9 d in blood cells (Figure 1, Table 3). In egg tissues, *λ* averaged 0.218±0.041 (SE) (*t_50_* = 3.2 d) and 0.116±0.020 (SE) (*t_50_* = 6.0 d) in albumen and yolk, respectively (Figure 2, Table 3). However, in yolk we found considerably differences in *λ* in accordance with pattern of yolk formation: turn-over rates were slowest (0.052±0.033 (SE)) for inner yolk (synthesized first), followed by intermediate yolk (0.128±0.034 (SE)) and, finally, most rapid (0.222±0.034 (SE)) in outer yolk (synthesized last; Figure 2 inset).

**Table 3:** Regression statistics of the exponential decay functions in δ^13^C of different tissues from female mallards after the switch from C4 to C3-based diet.

Tissue	a	*λ*	*δ(∞)*	*R^2^*	n
Plasma	6.32±0.37	0.162±0.033	−24.31±0.25	0.94	13
Blood cells	7.60±1.37	0.022±0.007	−26.1±1.41	0.95	26
Albumen	5.31±0.29	0.218±0.041	−22.98±0.21	0.97	13
Yolk – mean	6.02±0.38	0.116±0.020	−23.27±0.36	0.98	13
inner yolk	7.58±2.62	0.052±0.033	−25.12±2.73	0.90	13
intermediate yolk	6.23±0.59	0.128±0.034	−23.22±0.53	0.94	13
outer yolk	5.73±0.27	0.222±0.034	−22.80±0.18	0.98	13

Exponential decay is given with *δ(t) = δ(∞)+a•e^−λ• t^* wherein *δ(t)* is the isotope signature of the specific tissue at time *t* (days) since diet switch, δ(∞) is the isotopic composition of the tissues in equilibrium with the new C3-diet, *a* is the difference between *δ(0)* and *δ(∞)*, and *λ* is the turn-over rate. R^2^ and n are goodness of fit and the number of samples; coefficients are given as means ± SE.

## Discussion

Studies in stable isotope ecology are fundamentally underpinned by knowledge of how closely stable isotope ratios in various animal tissues reflect isotope ratios found either in the ambient environment [30] or in the consumed diet (so-called source-tissue discrimination, [6]), as well as how rapidly these tissues take-over the isotopic information of a changed environment or diet (so-called turnover rates and related tissue half-life estimates, [6], [17]). Here, we have determined stable isotope discrimination factors and turn-over rates in tissues of four herbivorous waterbird species with similar digestive adaptations to a low-protein and high-fibre content diet [31].

### Source-tissue discrimination

We verified that for both carbon and nitrogen, apparent discrimination factors between source and tissues show considerable differences between different tissues, even those formed of a similar material and/or within a similar time frame, such as the three keratinous tissues (flight and back feathers and claws). In contrast to studies spanning multiple foraging guilds [19], between-species differences appeared to be minimal or absent in our study of herbivorous waterbirds. Notably, source-tissue discrimination of feathers was positive (enriched) for adults but negative (depleted) for chick down. Chick down feathers aside, discrimination factors were larger for keratinous tissues than for blood cells or plasma both in carbon and nitrogen, as seen in other avian studies [6] [32].

The difference in apparent discrimination factors between flight and body feathers may have been confounded by the temporal dynamics of feather re-growth, which varies between different feather tracts. In waterfowl, wing feathers are moulted completely within few weeks only [33]. To facilitate such rapid feather synthesis birds may use both exogenous (diet) and endogenous (stored) resources [34]. By contrast, body feathers are moulted over substantially longer time periods, and claws are grown continuously throughout the annual cycle, suggesting potentially lower physiological stress for the individual. We cannot, however, exclude the possibility that some variation in discrimination factors may be a result of individual or species diet selectivity, given that we calculated discrimination factors from bulk diet to studied tissue. It is still possible that birds may have fed selectively during specific periods of the annual cycle, which could explain the interspecies differences in discrimination factors for back feathers or plasma.

Variability in apparent discrimination factors between different tissues is widely acknowledged [35] [36] [14] [6]; thought to be a result of different biochemical pathways and hence different sets of enzymes which can operate isotope-specifically [5]. Yet certain tissues that we would assume to have been produced by similar biochemical pathways (such as keratinous claws and feathers) showed variation in discrimination factors, particularly for nitrogen (Table 2). Any potential differences in keratin synthesis aside, such differences in discrimination factors may be attributed to specific routing of nutrients to and from body stores [21], which, in migrants, may vary throughout the annual cycle.

### Source-tissue discrimination across guilds

Given that discrimination depends on chemical composition of diet and its subsequent digestion, discrimination factors are expected to differ between foraging guilds. Indeed, marked differences between carnivorous and granivorous animals have previously been reported [19]. To further examine whether foraging guild may influence discrimination of carbon and nitrogen we complied available data on avian species studied to date (March 2011). We applied general linear mixed models to examine the effect of foraging guild (8 levels) and target tissue (10 levels, Supporting Material Table S2) on Δ^13^C or Δ^15^N, with study and species as random effects. We assumed that Δ^13^C of whole blood and blood cells were similar, given the overwhelming isotopic signal of blood cells on the isotope ratios in the blood as a whole [17]. Carbon discrimination differed between tissues (*P*<0.001) but not foraging guilds (*P* = 0.23), as did Δ^15^N (*P*<0.001 and *P* = 0.08 for tissue and foraging guild, respectively). We also tested whether our values for Δ^13^C or Δ^15^N in herbivorous waterfowl differed from those obtained for granivorous birds - the current surrogate values for herbivores - for blood cells and feathers (data for plasma, claws and chick down are not available for granivorous birds). There were no significant differences for Δ^13^C of blood cells or feathers (*P* = 0.87 and *P* = 0.57, respectively) or for Δ^15^N of blood cells (*P* = 0.11), but significantly different Δ^15^N of feathers (*P* = 0.01).

Within a foraging guild, similar Δ of same tissues can be expected if the individuals are in steady state [37]. Indeed, we show similarities in discrimination factors across herbivorous waterbirds within tissues. However, the species in our study are closely related members of the same family (Anatidae); a comparison of discrimination factors between non-related herbivorous species is lacking at present.

### Turn-over in blood and egg tissues

Diet switches occur frequently in the wild, when individuals travel (and feed) between isotopically distinct habitats [17]. Given that different tissues turn-over at different rates, it is then possible to infer an animal's diet or habitat preference over a range of temporal and spatial scales by examining isotopic composition of two or more of these tissues [38]. Yet conclusions derived from specific isotope patterns are critically underpinned by knowledge of the time needed to incorporate a specific pattern into the target tissue(s). For herbivorous waterbirds, we show a 6-fold difference in half-life time between blood plasma and blood cells (Table 1), underscoring the utility of these tissues for studies of diet switch dynamics ([17]).

Adding our estimate of δ^13^C to the available literature on blood cells and whole blood (Supporting Material Table S3), we found weak support for the notion that turnover of tissues is related to body mass scaled to the ¼ power [22] [20] such that *t_50_ = 5.27•body mass(g)^0.212^* (*r^2^* = 0.45, *P* = 0.01). Despite this relationship, considerable interspecies variation exists. In fact, the above relationship underestimates our empirically derived value for blood cell *t_50_* by approximately 30%, highlighting the need for species-specific estimates and a greater understanding of the mechanisms driving interspecies differences in tissue turnover before we can simply estimate turnover rates based on the body mass of an animal of interest.

We also show, for the first time that yolk and albumen of eggs laid following a diet switch show similar half-life dynamics as blood plasma; with albumen turning over slightly faster than yolk. In addition, we were able to quantify a within-yolk pattern of isotopic incorporation following a diet switch that was directly related to the pattern of yolk formation. This has two important implications: first, in laying females, digested nutrients appear to be readily routed to albumen and yolk formation. This is supported by the similarities between the rate of development/turnover in yolk and albumen and the turnover rate of blood plasma, the main carrier for metabolic products and pre-cursors for egg formation (Table 1). Secondly, yolk archives the isotopic pattern of food along a temporal gradient from inner- (oldest) to outer-most (newest) parts of the yolk. Thus, the isotopic pattern in yolk rather gives a snapshot in time of the diet composition, similar to keratin in feathers or claws, than a genuine turn-over driven by continuous supply of nutrients like blood. This pattern both extends the potential utility of egg tissues in studies of diet switch dynamics and emphasizes the importance of standardized yolk sampling techniques, as random yolk samples taken from raw eggs may vary in their age and isotopic composition.

Across all avian species studied to date some generalities regarding discrimination factors and tissue turn-over times can be made. Typically, however, a large amount of variation still remains unexplained.

## Supporting Information

Table S1 Data of stable carbon isotope ratios (*δ^13^C*) and stable nitrogen isotope ratios (*δ^15^N*) of various sources and tissues for calculation of discrimination factors. Data are given as means ± SD; n indicates sample size.
(DOC)

Table S2 Tissue-to-diet discrimination factors from avian species (until March 2011) for a range of body tissues.
(DOC)

Table S3 Average half-life of δ^13^C in tissues (blood cells, blood plasma, egg yolk and egg albumen) following an experimental diet switch for the avian species. When multiple studies on the same species were available, data were averaged for the analysis (see text).
(DOC)

## References

[1] Peterson BJ, Fry B (1987). Stable isotopes in ecosystem studies.. Annu Rev Ecol Syst.

[2] Bearhop S, Hilton GM, Votier SC, Waldron S (2004). Stable isotope ratios indicate that body condition in migrating passerines is influenced by winter habitat.. P Roy Soc B Biol Sci.

[3] Bocher P, Cherel Y, Hobson KA (2000). Complete trophic segregation between South Georgian and common diving petrels during breeding at Iles Kerguelen.. Mar Ecol -Prog Ser.

[4] Hobson KA, Hobson KA, Wassenaar LI (2008). Applying isotopic methods to tracking animal movements.. Tracking animal migration with stable isotopes.

[5] del Rio CM, Wolf N, Carleton SA, Gannes LZ (2009). Isotopic ecology ten years after a call for more laboratory experiments.. Biol Rev.

[6] Pearson SF, Levey DJ, Greenberg CH, del Rio CM (2003). Effects of elemental composition on the incorporation of dietary nitrogen and carbon isotopic signatures in an omnivorous songbird.. Oecologia.

[7] Fry B (2006). Stable isotope ecology.

[8] Gannes LZ, del Rio CM, Koch P (1998). Natural abundance variations in stable isotopes and their potential uses in animal physiological ecology.. Comp Biochem Phys A.

[9] Caut S, Angulo E, Courchamp F (2009). Variation in discrimination factors (Delta N-15 and Delta C-13): the effect of diet isotopic values and applications for diet reconstruction.. J Appl Ecol.

[10] McCutchan JH, Lewis WM, Kendall C, McGrath CC (2003). Variation in trophic shift for stable isotope ratios of carbon, nitrogen, and sulfur.. Oikos.

[11] Robbins CT, Felicetti LA, Sponheimer M (2005). The effect of dietary protein quality on nitrogen isotope discrimination in mammals and birds.. Oecologia.

[12] Williams CT, Buck CL, Sears J, Kitaysky AS (2007). Effects of nutritional restriction on nitrogen and carbon stable isotopes in growing seabirds.. Oecologia.

[13] Hobson KA, Clark RG (1992). Assessing avian diets using stable isotopes.1. Turnover of C-13 in tissues.. Condor.

[14] Bearhop S, Waldron S, Votier SC, Furness RW (2002). Factors that influence assimilation rates and fractionation of nitrogen and carbon stable isotopes in avian blood and feathers.. Physiol Biochem Zool.

[15] Bearhop S, Furness RW, Hilton GM, Votier SC, Waldron S (2003). A forensic approach to understanding diet and habitat use from stable isotope analysis of (avian) claw material.. Funct Ecol.

[16] Smith AD, Dufty AM (2005). Variation in the stable-hydrogen isotope composition of Northern goshawk feathers: Relevance to the study of migratory origins.. Condor.

[17] Klaassen M, Piersma T, Korthals H, Dekinga A, Dietz MW (2010). Single-point isotope measurements in blood cells and plasma to estimate the time since diet switches.. Funct Ecol.

[18] Robbins CT, Felicetti LA, Florin ST (2010). The impact of protein quality on stable nitrogen isotope ratio discrimination and assimilated diet estimation.. Oecologia.

[19] Hobson KA, Clark RG (1992). Assessing avian diets using stable isotopes. 2. Factors influencing diet-tissue fractionation.. Condor.

[20] Bauchinger U, McWilliams S (2009). Carbon turnover in tissues of a passerine bird: allometry, isotopic clocks, and phenotypic flexibility in organ size.. Physiol Biochem Zool.

[21] Dalerum F, Angerbjorn A (2005). Resolving temporal variation in vertebrate diets using naturally occurring stable isotopes.. Oecologia.

[22] Carleton SA, del Rio CM (2005). The effect of cold-induced increased metabolic rate on the rate of C-13 and N-15 incorporation in house sparrows (*Passer domesticus*).. Oecologia.

[23] Vanderklift MA, Ponsard S (2003). Sources of variation in consumer-diet delta N-15 enrichment: a meta-analysis.. Oecologia.

[24] Perga ME, Grey J (2010). Laboratory measures of isotope discrimination factors: comments on Caut, Angulo & Courchamp (2008,2009).. J Appl Ecol.

[25] Hahn S, Loonen MJJE, Klaassen M (2011). The reliance on distant resources for egg formation in high Arctic breeding barnacle geese.. J Avian Biol.

[26] Owen M (1975). Assessment of fecal analysis technique in waterfowl feeding studies.. J Wildl Mgmt.

[27] Sjögersten S, Kuijper DPJ, Van der Wal R, Loonen MJJE, Huiskes AHL (2010). Nitrogen transfer between herbivores and their forage species.. Polar Biol.

[28] Alisauskas RT, Ankney CD, Blatt BDJ, Afton AD, Anderson MG, Ankney CD, Johnson DH (1992). The cost of egg laying and its relationship to nutrient reserves in waterfowl.. Ecology and management of breeding waterfowl.

[29] Roudybush TE, Grau CR, Petersen MR, Ainley DG, Hirsch KV (1979). Yolk formation in some charadriiform birds.. Condor.

[30] Grossman EL, Ku T-L (1986). Oxygen and carbon isotope fractionation in biogenic aragonite: temperature effects.. Chemical Geology (Isotope Geoscience Section).

[31] Karasov WH (1990). Digestion in birds: chemical and physiological determinants and ecological implications.. Stud Avian Biol.

[32] Bugoni L, Mcgill RAR, Furness RW (2008). Effects of preservation methods on stable isotope signatures in bird tissues.. Rapid Commun Mass Sp.

[33] Kear J (1963). The agricultural importance of wild goose droppings.. The Wildfowl Trust Annual Report 1961–62.

[34] Fox AD, Hobson KA, Kahlert J (2009). Isotopic evidence for endogenous protein contributions to greylag goose *Anser anser* flight feathers.. J Avian Biol.

[35] Tieszen LL, Boutton TW, Tesdahl KG, Slade NA (1983). Fractionation and turnover of stable carbon isotopes in animal tissues - Implications for delta-C-13 analysis of diet.. Oecologia.

[36] Mizutani H, Kabaya Y, Wada E (1991). Nitrogen and carbon isotope compositions relate linearly in cormorant tissues and its diet.. Isotopenpraxis.

[37] Cherel Y, Hobson KA, Bailleul FR, Groscolas R (2005). Nutrition, physiology, and stable isotopes: New information from fasting and molting penguins.. Ecology.

[38] Inger R, Gudmundsson GA, Ruxton GD, Newton J, Colhoun K (2008). Habitat utilisation during staging affects body condition in a long distance migrant, *Branta bernicla hrota*: potential impacts on fitness?. J Avian Biol.

