# Supplementary material for: From Food to Offspring Down: Tissue-Specific Discrimination and Turn-Over of Stable Isotopes in Herbivorous Waterbirds and Other Avian Foraging Guilds
Source: PLoS One. 2012 Feb 1;7(2):e30242. doi: 10.1371/journal.pone.0030242 (PMC3270017; doi:10.1371/journal.pone.0030242)
Supplement: Table S1 — Data of stable carbon isotope ratios (δ13C) and stable nitrogen isotope ratios (δ15N) of various sources and tissues for calculation of discrimination factors. Data are given as means ± SD; n indicates sample size. (DOC) [file pone.0030242.s001.doc]

Table S1. Data of stable carbon isotope ratios (*δ^13^C*) and stable nitrogen isotope ratios (*δ^15^N*) of various sources and tissues for calculation of discrimination factors. Data are given as means ± SD; n indicates sample size.

| *Source* | *δ^13^C* | *δ^15^N* | *n* | *Tissue* | *δ^13^C* | *δ^15^N* | *n* | *Species* |
| --- | --- | --- | --- | --- | --- | --- | --- | --- |
| Food | -21.5±0.39 | 2.0±0.45 | 2* | plasma | -21.0±0.37 | 6.0±0.25 | 9 | Bewick Swan |
|  |  |  |  |  | -21.3±0.28 | 6.6±0.32 | 9 | Mallard |
|  |  |  |  | blood cells | -22.1±0.54 | 5.6±0.23 | 9 | Bewick Swan |
|  |  |  |  |  | -21.8±0.35 | 5.5±0.31 | 9 | Mallard |
|  |  |  |  | claws | -21.1±0.42 | 6.6±0.65 | 9 | Bewick Swan |
|  |  |  |  |  | -21.0±0.47 | 6.3±0.20 | 9 | Mallard |
|  |  |  |  | back feather | -19.9±0.98 | 6.5±0.53 | 9 | Bewick Swan |
|  |  |  |  |  | -21.1±0.37 | 6.8±0.55 | 9 | Mallard |
|  |  |  |  | primary feather | -20.8±0.77 | 7.3±0.40 | 9 | Bewick Swan |
|  |  |  |  |  | -20.3±0.31 | 7.1±0.19 | 9 | Mallard |
| Dropping | -29.3±0.76 | 5.4±1.56 | 44 | yolk | -26.8±0.27 | 8.2±2.24 | 4** | Pink-footed Goose |
| Albumen | -26.8±0.73 | 5.0±0.84 | 10 | down feather | -27.6±0.40 | 7.4±0.96 | 10 | Pink-footed Goose |
|  | -27.4±0.67 | 10.1±2.67 | 9 |  | -28.3±0.47 | 13.2±3.94 | 9 | Barnacle Goose |
| Yolk | -26.8±0.62 | 5.3±1.10 | 10 |  |  |  |  | Pink-footed Goose |
|  | -27.0±0.46 | 11.5±3.68 | 9 |  |  |  |  | Barnacle Goose |

* two random samples from diet mix, ** 10 follicles from four females
