# Supplementary material for: From Food to Offspring Down: Tissue-Specific Discrimination and Turn-Over of Stable Isotopes in Herbivorous Waterbirds and Other Avian Foraging Guilds
Source: PLoS One. 2012 Feb 1;7(2):e30242. doi: 10.1371/journal.pone.0030242 (PMC3270017; doi:10.1371/journal.pone.0030242)
Supplement: Table S2 — Tissue-to-diet discrimination factors from avian species (until March 2011) for a range of body tissues. (DOC) [file pone.0030242.s002.doc]

Table S2. Tissue-to-diet discrimination factors from avian species (until December 2010) for a range of body tissues.

| *Tissue* | *Foraging guild* | *Species* | *Scientific Name* | *Diet* |  |  | *Discrimination* | | *Remarks* | *Reference* |
| --- | --- | --- | --- | --- | --- | --- | --- | --- | --- | --- |
|  |  |  |  | *Composition* | *δ^13^C* | *δ^15^N* | *Δ δ^13^C* | *Δ δ^15^N* |  |  |
| *Blood* | | | | | | | | | |  |
|  | carnivore | Peregrine Falcon | *Falco peregrinus* | quail muscle |  |  | 0.20 | 3.30 |  | [1] |
|  |  | Great Skua | *Stercorarius skua* | fish (excl. Lipid) | -18.3 | 11.1 | 1.10 | 2.80 |  | [2] |
|  |  |  |  | fish (incl. Lipid) | -21.5 | 11.3 | 4.30 | 2.60 |  | [2] |
|  |  |  |  | Beef (incl. Lipid)† | -29.3 | 7.6 | 7.10 | 4.00 |  | [2] |
|  |  |  |  | Beef (excl. Lipid)† | -24.5 | 7.4 | 2.30 | 4.20 |  | [2] |
|  | carnivore/insectivore | Red Knot | *Calidris canutus* | fish (trout pellets) | -20.5 |  | 2.30 |  | cells only | [3] |
|  | granivore | Japanese Quail | *Coturnix japonica* | maize mix (C4) | -19.5 |  | -0.90 |  |  | [4] |
|  |  |  |  | wheat mix (C3) | -24.1 |  | 0.60 |  |  | [4] |
|  |  |  |  | turkey starter | -24.3 | 5.5 | 1.20 | 2.20 |  | [1] |
|  | herbivore | Mallard | *Anas platyrhynchos* | grain + chicken mash | -21.5 | 2.0 | -0.34 | 3.60 | cells only | This study |
|  |  | Canvasback | *Aythya valisineria* | grain mix | -24.0 | 4.1 | 1.45 | 2.98 |  | [5] |
|  |  | Bewick's swan | *Cygnus columbianus bewickii* | grain + chicken mash | -21.5 | 2.0 | -0.69 | 3.69 | cells only | This study |
|  | insectivore | Garden Warbler | *Sylvia borin* | fruit + insects | -25.8 | 6.5 | 1.70 | 2.40 |  | [6] |
|  |  | Dunlin | *Calidris alpina* | wheat/animal protein mix | -24.7 | 3.5 | 1.50 | 3.00 |  | [7] |
|  |  |  |  | Marine diet | -18.3 | 13.7 | 1.10 | 3.20 |  | [7] |
|  |  |  |  | wheat/animal protein mix | -24.7 | 3.5 | 1.30 | 2.90 |  | [7] |
|  | insectivore/frugivore | Yellow-rumped Warbler | *Dendroica coronata* | corn (C4) | -13.7 |  | -3.00 |  | cells only | [8] |
|  |  |  |  | sugar beet (C3) | -25.2 |  | 1.40 |  | cells only | [8] |
|  |  |  |  | fruit (80%) | -24.9 | 6.2 | -1.20 | 1.70 |  | [9] |
|  |  |  |  | insect (49%) | -26.7 | 6.1 | 1.50 | 1.80 |  | [9] |
|  |  |  |  | insect (73%) | -27.3 | 6.0 | 1.80 |  |  | [9] |
|  |  |  |  | insect (97%) | -27.9 | 6.1 | 2.20 | 2.70 |  | [9] |
|  |  | Red-throated Ant Tanager | *Habia fuscicauda* | Fruit (65%) mix | -21.7 | 3.6 | 2.20 | 2.60 |  | [10] |
|  | ominivore | American Crow | *Corvus bruchyrhynchos* | c3mix/c4 mix | -15.5 |  | 2.26 |  | cells only | [11] |
|  | piscivore | Rockhopper Penguin | *Eudyptes chrysocome* | fish | -19.5 | 10.5 | 0.02 | 2.72 |  | [12] |
|  |  |  |  | fish muscle | -19.9 | 11.4 | 0.46 | 1.86 |  | [12] |
|  |  | King Penguin | *Aptenodytes patagonicus* | fish | -18.2 | 12.6 | -0.80 | 2.07 |  | [12] |
|  |  |  |  | fish muscle | -18.4 | 13.4 | -0.61 | 1.23 |  | [12] |
|  |  | Ringbilled Gull | *Larus delawarensis* | fish (perch) | -24.3 | 14.2 | -0.30 | 3.10 |  | [1] |
|  |  |  |  |  |  |  |  |  |  |  |
| *Plasma* | | | | | | | | | |  |
|  | carnivore/insectivore | Red Knot | *Calidris canutus* | fish (trout pellets) | -20.5 |  | 0.80 |  |  | [3] |
|  | herbivore | Mallard | *Anas platyrhynchos* | grain + chicken mash | -21.5 | 2.0 | 0.18 | 4.67 |  | This study |
|  |  | Bewick's swan | *Cygnus columbianus bewickii* | grain + chicken mash | -21.5 | 2.0 | 0.42 | 4.04 |  | This study |
|  | insectivore | Dunlin | *Calidris alpina* | wheat + animal protein | -24.7 | 3.5 | 0.50 | 3.30 |  | [7] |
|  | insectivore/frugivore | Yellow-rumped Warbler | *Dendroica coronata* | fruit (80%) | -24.9 | 6.2 | -1.50 | 2.50 |  | [9] |
|  |  |  |  | insect (49%) | -26.7 | 6.1 | -0.20 | 2.60 |  | [9] |
|  |  |  |  | insect (73%) | -27.3 | 6.0 | 0.20 | 2.80 |  | [9] |
|  |  |  |  | insect (97%) | -27.9 | 6.1 | 0.61 | 3.00 |  | [9] |
|  |  |  |  | corn (C4) | -13.7 |  | -2.60 |  |  | [8] |
|  |  |  |  | sugar beet (C3) | -25.2 |  | 0.10 |  |  | [8] |
|  | ominivore | American Crow | *Corvus bruchyrhynchos* | c3mix/c4 mix | -15.5 |  | 0.33 |  |  | [11] |
|  |  |  |  |  |  |  |  |  |  |  |
| *Liver* | | | | | | | | | |  |
|  | granivore | Japanese Quail | *Coturnix japonica* | maize mix (C4) | -19.5 |  | -1.00 |  |  | [4] |
|  |  |  |  | wheat mix (C3) | -24.1 |  | 0.65 |  |  | [4] |
|  |  |  |  | turkey starter | -24.3 | 5.5 | 0.20 | 2.30 |  | [1] |
|  |  | Domestic chicken | *Gallus gallus* | turkey starter | -19.9 | 4.7 | 0.40 | 1.70 |  | [1] |
|  | insectivore | Dunlin | *Calidris alpina* | wheat + animal protein | -24.7 | 3.5 | 1.10 | 4.00 |  | [7] |
|  | piscivore | Great Cormorant | *Phalacrocorax carbo* | fish (mackerel) | -20.2 | 9.5 | 1.30 | 2.30 |  | [13] |
|  |  | Ringbilled Gull | *Larus delawarensis* | fish (perch) | -24.3 | 14.2 | -0.40 | 2.70 |  | [1] |
|  |  |  |  |  |  |  |  |  |  |  |
| *Muscle* | | | | | | | | | |  |
|  | carnivore | Upland buzzard | *Buteo hemilasius* | mammal (Plateau pikas) | -26.0 | 5.81 | 1.03 | 2.11 |  | [14] |
|  | granivore | Japanese Quail | *Coturnix japonica* | maize mix (C4) | -19.5 |  | 0.00 |  |  | [4] |
|  |  |  |  | wheat mix (C3) | -24.1 |  | 0.75 |  |  | [4] |
|  |  |  |  | turkey starter | -24.3 | 5.5 | 1.10 | 1.00 |  | [1] |
|  |  | Domestic chicken | *Gallus gallus* | turkey starter | -19.9 | 4.7 | 0.30 | 0.20 |  | [1] |
|  | insectivore | Dunlin | *Calidris alpina* | wheat + animal protein | -24.7 | 3.5 | 1.90 | 3.10 |  | [7] |
|  | piscivore | Great Cormorant | *Phalacrocorax carbo* | fish (mackerel) | -20.2 | 9.5 | 2.10 | 2.40 |  | [13] |
|  |  | Ringbilled Gull | *Larus delawarensis* | fish (perch) | -24.3 | 14.2 | 0.30 | 1.40 |  | [1] |
|  |  |  |  |  |  |  |  |  |  |  |
| *Bone collagen* | | | | | | | | | |  |
|  | granivore | Japanese Quail | *Coturnix japonica* | maize mix (C4) | -19.5 |  | -0.30 |  |  | [4] |
|  |  |  |  | wheat mix (C3) | -24.1 |  | 2.95 |  |  | [4] |
|  |  |  |  | turkey starter | -24.3 | 5.5 | 2.70 | 2.50 |  | [1] |
|  |  | Domestic chicken | *Gallus gallus* | turkey starter | -19.9 | 4.7 | 0.80 | 1.50 |  | [1] |
|  | piscivore | Ringbilled Gull | *Larus delawarensis* | fish (perch) | -24.3 | 14.2 | 2.60 | 3.10 |  | [1] |
|  |  |  |  |  |  |  |  |  |  |  |
| *Claw* | | | | | | | | | |  |
|  | herbivore | Mallard | *Anas platyrhynchos* | grain + chicken mash | -21.5 | 2.0 | 0.46 | 4.35 |  | This study |
|  |  | Bewick's swan | *Cygnus columbianus bewickii* | grain + chicken mash | -21.5 | 2.0 | 0.34 | 4.61 |  | This study |
|  |  |  |  |  |  |  |  |  |  |  |
| *Feather* | | | | | | | | | |  |
|  | carnivore | American White Ibis | *Eudocimus albus* | corn (C4) | -22.4 | 3.6 | 2.50 | 4.30 |  | [15] |
|  |  | Scarlet Ibis | *Eudocimus ruber* | corn (C4) | -23.2 | 3.5 | 3.80 | 4.50 |  | [15] |
|  |  | Peregrine Falcon | *Falco peregrinus* | quail muscle |  |  | 2.10 | 2.70 |  | [1] |
|  |  | Brown Skua | *Stercorarius antarctica* | bird (prion; from feather) |  |  | 0.40 | 3.00 |  | [16] |
|  |  | Great Skua | *Stercorarius skua* | fish (incl. Lipid) | -21.5 | 11.3 | 5.30 | 4.40 |  | [2] |
|  |  |  |  | fish (excl. Lipid) | -18.3 | 11.1 | 2.10 | 4.60 |  | [2] |
|  |  |  |  | beef (incl. Lipid)† | -29.3 | 7.6 | 7.00 | 4.80 |  | [2] |
|  |  |  |  | beef (excl. Lipid)† | -24.5 | 7.4 | 2.20 | 5.00 |  | [2] |
|  | granivore | Japanese Quail | *Coturnix japonica* | turkey starter | -24.3 | 5.5 | 1.40 | 3.10 |  | [1] |
|  |  | Domestic chicken | *Gallus gallus* | turkey starter | -19.9 | 4.7 | -0.40 | 1.10 |  | [1] |
|  | herbivore | Mallard | *Anas platyrhynchos* | grain + chicken mash | -21.5 | 2.0 | 0.39 | 4.89 | back | This study |
|  |  |  |  | grain + chicken mash | -21.5 | 2.0 | 1.12 | 5.11 | primary | This study |
|  |  | Bewick's swan | *Cygnus columbianus bewickii* | grain + chicken mash | -21.5 | 2.0 | 1.52 | 4.60 | back | This study |
|  |  |  |  | grain + chicken mash | -21.5 | 2.0 | 0.64 | 5.39 | primary | This study |
|  | insectivore | Garden Warbler | *Sylvia borin* | fruit + insects | -25.8 | 6.5 | 2.70 | 4.00 |  | This study |
|  | insectivore/frugivore | Yellow-rumped Warbler | *Dendroica coronata* | fruit (80%) | -24.9 | 6.2 | 1.90 | 3.20 |  | [6] |
|  |  |  |  | insect (97%) | -27.9 | 6.1 | 4.30 | 3.50 |  | [9] |
|  | ominivore | American Crow | *Corvus bruchyrhynchos* | maize mix (C4) | -20.1 |  | 3.50 |  |  | [4] |
|  |  |  |  | wheat mix (C3) | -23.7 |  | 4.40 |  |  | [4] |
|  | piscivore | European Shag | *Phalacrocorax aristotelis* | fish (sandeel) | -17.5 | 7.9 | 2.00 | 3.60 |  | [17] |
|  |  | Great Cormorant | *Phalacrocorax carbo* | fish (sprat) | -17.5 | 11.5 | 2.60 | 4.90 |  | [17] |
|  |  |  |  | fish (mackerel) | -20.2 | 9.5 | 3.60 | 3.60 |  | [13] |
|  |  |  |  | fish (mackerel) | -20.1 | 9.5 | 3.80 | 3.70 |  | [15] |
|  |  | Great white egret | *Ardea alba* | fish (saurel) | -18.3 | 9.7 | 3.10 | 3.90 |  | [15] |
|  |  | Grey Heron | *Ardea cinerea* | fish (saurel) | -18.3 | 9.7 | 3.40 | 4.30 |  | [15] |
|  |  | Nankeen night Heron | *Nycticorax caledonicus* | fish (saurel) | -18.3 | 9.7 | 3.20 | 4.20 |  | [15] |
|  |  | Rockhopper Penguin | *Eudyptes chrysocome* | fish | -19.5 | 10.5 | 0.11 | 4.40 |  | [12] |
|  |  |  |  | fish muscle | -19.9 | 11.4 | 0.55 | 3.53 |  | [12] |
|  |  | Humbolt penguin | *Spheniscus humboldti* | fish (anchovy) | -18.8 | 7.8 | 2.90 | 4.80 |  | [15] |
|  |  | King Penguin | *Aptenodytes patagonicus* | fish | -18.2 | 12.6 | 0.70 | 3.49 |  | [12] |
|  |  |  |  | fish muscle | -18.4 | 13.4 | 0.26 | 2.65 |  | [12] |
|  |  | Goosander | *Mergus merganser* | fish (salmon parr) | -23.1 | 7.2 | 2.40 | 4.20 |  | [17] |
|  |  | Black-tailed Gull | *Larus crassirostris* | fish (saurel) | -18.3 | 9.7 | 3.60 | 5.30 |  | [15] |
|  |  | Ringbilled Gull | *Larus delawarensis* | fish (perch) | -24.3 | 14.2 | 0.20 | 3.00 |  | [1] |
|  |  | Artic Tern | *Sterna paradisaea* | fish (sandeel) | -16.9 | 8.3 | 2.10 | 3.40 |  | [16] |
|  |  | Common Guillemot | *Uria aalge* | fish (sandeel) | -16.9 | 8.3 | 1.00 | 3.30 |  | [16] |
|  |  |  |  | fish muscle | -19.9 | 13.4 | 2.50 | 3.60 | body | [18] |
|  |  |  |  | fish muscle | -19.9 | 13.4 | 1.90 | 3.70 | primary | [18] |
|  | piscivore/planktivore | Broad-billed Prion | *Pachyptila vittata* | zooplankton | -20.1 | 6.5 | 2.50 | 4.30 |  | [16] |
|  | planktivore | Chilean Flamingo | *Phoenicopterus chilensis* | corn (C4) | -22.1 | 2.9 | 3.60 | 5.60 |  | [15] |
|  |  |  |  |  |  |  |  |  |  |  |
| *Albumen* | | | | | | | | | |  |
|  | carnivore | Prairie Falcon | *Falco mexicanus* | quail muscle | -23.4 | 4.2 | 0.90 | 3.10 |  | [19] |
|  |  | Peregrine Falcon | *Falco peregrinus* | quail muscle | -23.4 | 4.2 | 0.90 | 3.10 |  | [19] |
|  |  | Gyr Falcon | *Falco rusticolus* | quail muscle | -23.4 | 4.2 | 0.80 | 3.30 |  | [19] |
|  | granivore | Japanese Quail | *Coturnix japonica* | turkey starter | -24.0 | 2.8 | 1.60 | 2.40 |  | [19] |
|  | herbivore | Mallard | *Anas platyrhynchos* | grain + chicken mash | -24.4 | 3.6 | 1.40 | 2.90 |  | [19] |
|  |  |  |  |  |  |  |  |  |  |  |
| *Yolk* | | | | | | | | | |  |
|  | carnivore | Prairie Falcon | *Falco mexicanus* | quail muscle | -23.4 | 4.2 | -1.40 | 3.50 |  | [19] |
|  |  |  |  | quail muscle | -23.4 | 4.2 | 0.10 | 3.50 | excl. lipids | [19] |
|  |  | Peregrine Falcon | *Falco peregrinus* | quail muscle | -23.4 | 4.2 | -2.20 | 3.30 |  | [19] |
|  |  |  |  | quail muscle | -23.4 | 4.2 | 0.00 | 3.50 | excl. lipids | [19] |
|  |  | Gyr Falcon | *Falco rusticolus* | quail muscle | -23.4 | 4.2 | -1.80 | 3.10 |  | [19] |
|  |  |  |  | quail muscle | -23.4 | 4.2 | 0.10 | 3.60 | excl. lipids | [19] |
|  | granivore | Japanese Quail | *Coturnix japonica* | turkey starter | -24.0 | 2.8 | 0.10 | 3.40 |  | [19] |
|  |  |  |  | turkey starter | -24.0 | 2.8 | -1.10 | 3.40 |  | [19] |
|  | herbivore | Mallard | *Anas platyrhynchos* | Wheat/pellet mix | -24.4 | 3.6 | 0.05 | 3.15 |  | [19] |
|  |  |  |  | Wheat/pellet mix | -24.4 | 3.6 | -1.35 | 3.20 |  | [19] |
|  |  | Pink-footed Goose | *Anser brachyrhynchos* | grass (from droppings) | -29.3 | 5.4 | 2.5 | 2.8 | excl. lipids | This study |
|  |  |  |  |  |  |  |  |  |  |  |
| *Chick Down Feathers* | | | | | | | | | |  |
|  | herbivore | Pink-footed Goose | *Anser brachyrhynchos* | albumen |  |  | -0.82 | 2.40 |  | This study |
|  |  |  |  | yolk (excl. lipids) |  |  | -0.83 | 2.10 |  | This study |
|  |  | Barnacle Goose | *Branta leucopsis* | albumen |  |  | -0.89 | 3.09 |  | This study |
|  |  |  |  | yolk (excl. lipids) |  |  | -1.33 | 1.68 |  | This study |
|  | ominivore | Black-headed Gull | *Larus ridibundus* | yolk |  |  | 3.10 |  |  | [20] |

† Excluded from statistical analysis because the authors suggested these individuals may not have been at isotopic equilibium with their diet at the time the discrimination estimates were made.

References

1. Hobson KA, Clark RG (1992) Assessing avian diets using stable isotopes: 2. Factors influencing diet-tissue fractionation. Condor 94: 189-197.

2. Bearhop S, Waldron S, Votier SC, Furness RW (2002) Factors that influence assimilation rates and fractionation of nitrogen and carbon stable isotopes in avian blood and feathers. Physiol Biochem Zool 75: 451-458.

3. Klaassen M, Piersma T, Korthals H, Dekinga A, Dietz MW (2010) Single-point isotope measurements in blood cells and plasma to estimate the time since diet switches. Funct Ecol 24: 796-804.

4. Hobson KA, Clark RG (1992) Assessing avian diets using stable isotopes: 1. Turnover of C-13 in tissues. Condor 94: 181-188.

5. Haramis GM, Jorde DG, Macko SA, Walker JL (2001) Stable-isotope analysis of Canvasback winter diet in upper Chesapeake Bay. Auk 118: 1008-1017.

6. Hobson KA, Bairlein F (2003) Isotopic fractionation and turnover in captive Garden Warblers (Sylvia borin): implications for delineating dietary and migratory associations in wild passerines. Can J Zool 81: 1630-1635.

7. Evans Ogden LJ, Hobson KA, Lank DB (2004) Blood isotopic (delta C-13 and delta N-15) turnover and diet-tissue fractionation factors in captive Dunlin (Calidris alpina pacifica). Auk 121: 170-177.

8. Podlesak DW, McWilliams SR, Hatch KA (2005) Stable isotopes in breath, blood, feces and feathers can indicate intra-individual changes in the diet of migratory songbirds. Oecologia 142: 501-510.

9. Pearson SF, Levey DJ, Greenberg CH, del Rio CM (2003) Effects of elemental composition on the incorporation of dietary nitrogen and carbon isotopic signatures in an omnivorous songbird. Oecologia 135: 516-523.

10. Herrera LG, Reyna JC (2007) Stable carbon and nitrogen isotopic discrimination in whole blood of red-throated ant tanagers Habia fuscicauda. J Ornithol 148: 235-240.

11. Hobson KA, Clark RG (1993) Turnover of C-13 in cellular and plasma fractions of blood - implications for non-destructive sampling in avian dietary studies. Auk 110: 638-641.

12. Cherel Y, Hobson KA, Hassani S (2005) Isotopic discrimination between food and blood and feathers of captive penguins: Implications for dietary studies in the wild. Physiol Biochem Zool 78: 106-115.

13. Mizutani H, Kabaya Y, Wada E (1991) Nitrogen and carbon isotope compositions relate linearly in cormorant tissues and its diet. Isotopenpraxis 27: 166-168.

14. Li LX, Yi XF, Li MC, Zhang XA (2004) Analysis of diets of upland buzzards using stable carbon and nitrogen isotopes. Israel J Zool 50: 75-85.

15. Mizutani H, Fukuda M, Kabaya Y (1992) C-13 enrichment and N-15 enrichment factors of feathers of 11 species of adult bids. Ecology 73: 1391-1395.

16. Thompson DR, Furness RW (1995) Stable-isotope ratios of carbon and nitrogen in feathers indicate seasonal dietary shifts in Northern Fulmars. Auk 112: 493-498.

17. Bearhop S, Thompson DR, Waldron S, Russell IC, Alexander G, et al. (1999) Stable isotopes indicate the extent of freshwater feeding by cormorants Phalacrocorax carbo shot at inland fisheries in England. J Appl Ecol 36: 75-84.

18. Becker BH, Newman SH, Inglis S, Beissinger SR (2007) Diet-feather stable isotope (delta N-15 and delta C-13) fractionation in Common Murres and other seabirds. Condor 109: 451-456.

19. Hobson KA (1995) Reconstructing avian diets using stable-carbon and nitrogen isotope analysis of egg components - patterns of isotopic fractionation and turnover. Condor 97: 752-762.

20. Klaassen M, Baarspul T, Dekkers T, van Tienen P (2004) The relationship between carbon stable isotope ratios of hatchling down and egg yolk in Black-headed Gulls. J Field Ornithol 75: 196-199.
