# Supplementary material for: From Food to Offspring Down: Tissue-Specific Discrimination and Turn-Over of Stable Isotopes in Herbivorous Waterbirds and Other Avian Foraging Guilds
Source: PLoS One. 2012 Feb 1;7(2):e30242. doi: 10.1371/journal.pone.0030242 (PMC3270017; doi:10.1371/journal.pone.0030242)
Supplement: Table S3 — Average half-life of δ13C in tissues (blood cells, blood plasma, egg yolk and egg albumen) following an experimental diet switch for the avian species. When multiple studies on the same species were available, data were averaged for the analysis (see text). (DOC) [file pone.0030242.s003.doc]

Table S3. Average half-life of δ^13^C in tissues (blood cells, blood plasma, egg yolk and egg albumen) following an experimental diet switch for the avian species. When multiple studies on the same species were available, data were averaged for the analysis (see text).

| *Species* | *Scientific name* | *Body mass (g)* |  | *Half-life estimate* | | | | | *Reference* |
| --- | --- | --- | --- | --- | --- | --- | --- | --- | --- |
|  |  |  |  | *Blood* | | *Plasma* | *Yolk* | *Albumen* |  |
|  |  |  |  |  |  |  |  |  |  |
| Yellow-rumped Warbler | *Dendroica coronata* | 12.5 | a | 4.5 |  | 0.5 |  |  | [1] |
| Yellow-rumped Warbler | *Dendroica coronata* | 12.5 | a | 10.8 | * | 1.1 |  |  | [2] |
| Zebra Finch | *Taeniopygia guttata* | 16.0 |  | 13.4 | * |  |  |  | [3] |
| Garden warbler | *Sylvia borin* | 20.0 |  | 5.4 |  |  |  |  | [4] |
| House Sparrow | *Passer domesticus* | 22.0 |  | 15.8 | * |  |  |  | [5] |
| House Sparrow | *Passer domesticus* | 23.0 |  | 19.3 | * | 3.3 |  |  | [6] |
| Dunlin | *Calidris alpina* | 55.5 |  | 11.2 |  |  |  |  | [7] |
| Red Knot | *Calidris canutus* | 148.0 |  | 15.1 | * | 6 |  |  | [8] |
| Japanese Quail | *Coturnix japonica* | 190.0 | a | 11.4 |  |  | 4.5 | 2.3 | [9, 10] |
| American Crow | *Corvus brachyrhynchos* | 416.0 |  | 29.8 | * | 2.9 |  |  | [11] |
| Mallard | *Anas platyrhynchos* | 980.0 |  | 31.9 | * | 4.3 | 6.0 | 3.2 | This study |
| Great Skua | *Stercorarius skua* | 1220.0 |  | 15.7 |  |  |  |  | [12] |
| Canvasback | *Aythya valisineria* | 1248.0 | a | 22.8 |  |  |  |  | [13] |

* After the blood half-life estimate indicates that turnover was calculated for blood cells rather than whole blood

^a^ Body masses not given in the reference, were obtained from [5]

*References*

1. Pearson SF, Levey DJ, Greenberg CH, del Rio CM (2003) Effects of elemental composition on the incorporation of dietary nitrogen and carbon isotopic signatures in an omnivorous songbird. Oecologia 135: 516-523.

2. Podlesak DW, McWilliams SR, Hatch KA (2005) Stable isotopes in breath, blood, feces and feathers can indicate intra-individual changes in the diet of migratory songbirds. Oecologia 142: 501-510.

3. Bauchinger U, McWilliams S (2009) Carbon turnover in tissues of a passerine bird: allometry, isotopic clocks, and phenotypic flexibility in organ size. Physiol Biochem Zool 82: 787-797.

4. Hobson KA, Bairlein F (2003) Isotopic fractionation and turnover in captive Garden Warblers (Sylvia borin): implications for delineating dietary and migratory associations in wild passerines. Can J Zool 81: 1630-1635.

5. Carleton SA, Martínez del Rio C (2005) The effect of cold-induced increased metabolic rate on the rate of C-13 and N-15 incorporation in house sparrows (Passer domesticus). Oecologia 144: 226-232.

6. Carleton SA, Kelly L, Anderson-Sprecher R, del Rio CM (2008) Should we use one-, or multi-compartment models to describe C-13 incorporation into animal tissues? Rapid Commun Mass Sp 22: 3008-3014.

7. Evans Ogden LJ, Hobson KA, Lank DB (2004) Blood isotopic (delta C-13 and delta N-15) turnover and diet-tissue fractionation factors in captive Dunlin (Calidris alpina pacifica). Auk 121: 170-177.

8. Klaassen M, Piersma T, Korthals H, Dekinga A, Dietz MW (2010) Single-point isotope measurements in blood cells and plasma to estimate the time since diet switches. Funct Ecol 24: 796-804.

9. Hobson KA, Clark RG (1992) Assessing avian diets using stable isotopes: 1. Turnover of C-13 in tissues. Condor 94: 181-188.

10. Hobson KA (1995) Reconstructing avian diets using stable-carbon and nitrogen isotope analysis of egg components - patterns of isotopic fractionation and turnover. Condor 97: 752-762.

11. Hobson KA, Clark RG (1993) Turnover of C-13 in cellular and plasma fractions of blood - implications for non-destructive sampling in avian dietary studies. Auk 110: 638-641.

12. Bearhop S, Waldron S, Votier SC, Furness RW (2002) Factors that influence assimilation rates and fractionation of nitrogen and carbon stable isotopes in avian blood and feathers. Physiol Biochem Zool 75: 451-458.

13. Haramis GM, Jorde DG, Macko SA, Walker JL (2001) Stable-isotope analysis of Canvasback winter diet in upper Chesapeake Bay. Auk 118: 1008-1017.
